# Evaluation of Different Oligonucleotide Base Substitutions at CpG Binding sites in Multiplex Bisulfite-PCR sequencing

**DOI:** 10.1038/srep45096

**Published:** 2017-03-22

**Authors:** Jennifer Lu, Kelin Ru, Ida Candiloro, Alexander Dobrovic, Darren Korbie, Matt Trau

**Affiliations:** 1 Centre for Personalised Nanomedicine, Australian Institute for Nanoengineering and Biotechnology, University of Queensland, Brisbane, Australia; 2 Translational Genomics and Epigenomics Laboratory, Olivia Newton-John Cancer Research Institute, Melbourne, Victoria, 8006, Australia; 3 Department of Pathology, University of Melbourne, Parkville, Victoria, 3010, Australia; 4 School of Cancer Medicine, La Trobe University, Bundoora, Victoria, 3084, Australia; 5 Peter MacCallum Cancer Center, Parkville, 3010, Australia

## Abstract

Multiplex bisulfite-PCR sequencing is a convenient and scalable method for the quantitative determination of the methylation state of target DNA regions. A challenge of this application is the presence of CpGs in the same region where primers are being placed. A common solution to the presence of CpGs within a primer-binding region is to substitute a base degeneracy at the cytosine position. However, the efficacy of different substitutions and the extent to which bias towards methylated or unmethylated templates may occur has never been evaluated in bisulfite multiplex sequencing applications. In response, we examined the performance of four different primer substitutions at the cytosine position of CpG’s contained within the PCR primers. In this study, deoxyinosine-, 5-nitroindole-, mixed-base primers and primers with an abasic site were evaluated across a series of methylated controls. Primers that contained mixed- or deoxyinosine- base modifications performed most robustly. Mixed-base primers were further selected to determine the conditions that induce bias towards methylated templates. This identified an optimized set of conditions where the methylated state of bisulfite DNA templates can be accurately assessed using mixed-base primers, and expands the scope of bisulfite resequencing assays when working with challenging templates.

DNA methylation in higher eukaryotes occurs as a result of the addition of a methyl group to the carbon-5 position of cytosine-guanine (CpG) dinucleotides to form 5-methylcytosine. Due to the critical role of DNA methylation in a number of biological functions (e.g. X-chromosome inactivation, genomic imprinting and regulation of gene expression)^1^,^2^,^3^,^4^, a number of technologies have been developed to study and characterise the methylation patterns at a locus-specific level (e.g. DNA melting curve analysis, PCR-based methods). A common feature of these applications is the use of bisulfite modification^5^,^6^,^7^, wherein unmethylated cytosines are selectively deaminated to uracils and participate in subsequent PCR amplifications as thymines but 5-methylcytosines are left unaltered, which allows the visualisation of this epigenetic change using a variety of applications^8^,^9^. As such, the bisulfite-conversion process results in a pool of templates which has cytosine/thymine degeneracy at the cytosine positions of CpG dinucleotides, and PCR primers designed against these regions must therefore be able to bind to both cytosines (methylated) and thymines (unmethylated) when unbiased amplification is sought. However, under non-optimized conditions a PCR primer that binds to a mixed-template CpG site may differentially amplify the methylated or unmethylated DNA strand, thereby inducing bias in the data and an inaccurate representation of the methylation state of the amplified products^10^,^11^.

Dealing with this potential bias represents a major challenge in bisulfite PCR assay design. The use of ‘CpG-free’ primers (i.e. primers that do not bind to a region with CpG dinucleotides) has been considered to produce ‘non–preferential’ amplification of bisulfite-converted^8^. However, this method is not always feasible, as most regions of interest are CpG-rich and the use of CpG-primers is often unavoidable. Although the use of ‘modified primers’ (i.e. primers with inclusion of modified bases) have been reported in a variety of bisulfite applications (data not shown), their efficacy and the extent to which methylated templates are preferentially amplified has never been evaluated in multiplex bisulfite PCR applications^12^.

In this study, we undertook a series of experiments to study the effect of PCR primers which anneals to a region containing a CpG site, and the effect of substituting a specialised modified base at this position in the oligonucleotide within the context of multiplex bisulfite PCR sequencing. Four modifications: deoxyinosine-, 5-nitroindole-, mixed- (where Y contains a ‘mixture’ of cytosines and thymines at the cytosine site, and R contains a mixture of guanines and adenines at the guanine site) and primers with an abasic site were selected based on their reported stabilities in previous studies^13^,^14^,^15^. The effects of selected modified bases using a number of different conditions were examined, with a focus on the use of different primer substitutions, position of the modified base within the primer, the impact of having a modification in only one primer verses having a modification in both the forward and reverse primers, as well as the optimal conditions under which to perform bisulfite multiplex PCR when using some of these modified bases.

## Results and Discussion

### Assessment of amplification fidelity of modified primers in standard bisulfite-PCR

Four different oligonucleotide modifications were selected for multiplex bisulfite PCR experiments. The abasic modification acts as a space in the primer, and is often used to study the damage and repair mechanisms in DNA^16^. For mixed-base primers, the modified base Y or R was used depending on the strand orientation of the template where Y (which represents C and T) was used for primers targeting the sense strand, while R (which represents G and A) was used for primers targeting the anti-sense strand^17^. The universal bases deoxyinosine^15^ and 5-nitroindole^13^,^18^,^19^, which have the ability to bind to any of the four DNA bases were also included, although the status of deoxyinosine as a universal base has been debated due to its preferential binding to cytosines^15^.

To isolate the effects of each modification, sets of identical primers were ordered which differed only at the cytosine position of the CpG dinucleotide-binding site contained within the PCR primer. The functionality of these assays was then assessed using a standard singleplex bisulfite PCR, with the amplified products visualised using gel electrophoresis (summary of the results listed in Table 1, with representative gel images in Supp Figure 1). Additionally, a panel of ‘CpG-free’ primers (i.e. the region where primers are designed have no CpG dinucleotides) were also included as control primers to ensure the PCR conditions used could generate suitable amplified products. During subsequent multiplexing analysis, ‘CpG-free’ control primers were used in each multiplex pool to ensure the results generated from primers with modifications were not as an artefact of the preparation of the sequencing libraries.

Under singleplex conditions, all three of the ‘CpG-free’ control primer pairs were observed to successfully produce amplicons of the predicted size, as well as primer pairs which contained either deoxyinosine or degenerate base substitutions (Supp Figure 1). In contrast, 50% (3 out of 6) of the assays which contained either the 5-nitroindole or the abasic modification failed to generate any observable product. To ensure the lack of amplification of the modified primers was not due to poor manufacture, shipment or handling, the complete set of primers were reordered from a different oligonucleotide manufacturer and screened using the same protocol, but results obtained from the second screen was identical to the first (data not shown), which indicated that the poor performance of the 5-nitroindole and abasic assays was due to the modification itself.

It was hypothesized that the poor performance of bisulfite PCR assays containing either 5-nitroindole or an abasic site could be related to the position of the modification within the primer binding site. In particular, modifications near the centre of the oligonucleotides was predicted to be more deleterious to amplification than those with modified bases positioned towards the 5′ terminus of the primer(s). To test this, a second panel of assays were designed with the modification placed either near the centre of the primer, or towards the 5′ end. Similar to the first screen, the amplification fidelity of each primer pair was assessed in a singleplex bisulfite PCR followed by gel electrophoresis (summary of the results listed in Table 2, with corresponding gel images in Supp Figure 2). Based on this result, it was concluded that the position of the modification did not affect amplicon formation when using oligonucleotides containing either the deoxyinosine or mixed-base modifications; however, primers with 5-nitroindole or an abasic site near the centre of the primer would result in failed assays, which was only partially offset by shifting the position of the modification towards the 5′ end of the oligonucleotide.

### Assessing bisulfite-PCR primers with modified bases in multiplex resequencing

**Figure 1:**
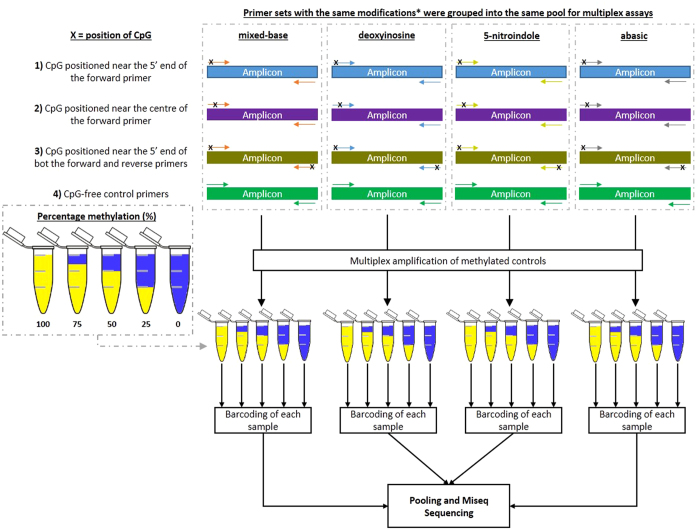
Outline of Modified Primers Study. Outline of the molecular structures of the different modified bases used in this study. Four different groups of base modifications were used in this study to compensate for the change in methylation statues of the cytosine site at CpG dinucleotides. Abasic modifications acts as a space in the primer and is commonly used to study the damage and repair mechanism in DNA. For mixed- bases, the modified base R or Y was used depending on the strand orientation of the template (i.e. Y (C and T) was used for sense strand, while R (G and A) was used for the anti-sense strand). The universal bases deoxyinosine and 5-nitroindole has the ability to bind to any of the four DNA bases and therefore was also included in this assay. Each primer pool was screened against a series of graduated methylated controls in order to observe the sensitivity of the primer pairs in reporting the different methylated levels within each control sample. Primers with the same modifications were mixed into the same pool during library preparation and additional CpG-free primer controls were included into each pool as an internal assay control. Each primer pool was used to screen a series of methylated controls (100%, 75%, 50%, 25 and 0% methylation) prior to barcoding of each sample, followed by pooling and sequencing of combined samples.

To determine if the inclusion of any of the four modifications in a PCR primer could induce bias in multiplex bisulfite PCR resequencing, all assays which successfully generated products in the singleplex PCR screen were combined into pools for multiplex screening. In brief, assays with the same base modification were grouped into the same multiplex pool regardless of the position or number of modifications in the primer pair (outline of multiplex experiment outlined in Fig. 1). Additionally, an identical set of ‘CpG-free’ control primers were also added to every multiplex pool as an internal control to identify the baseline methylation value for that sample. Different amplicons were differentiated by the addition of unique barcodes to each amplicon prior to pooling of products for final sequencing. Comparison of the methylation value of the CpG-free primers to the modified primers contained within the same multiplex reaction would then identify if a significant bias had been introduced as a result of a particular modification. Using this experimental setup, each primer pool was then assayed against five methylated controls (100% 75%, 50%, 25 and 0%), and the experimental baseline of each sample was determined by calculating the distribution of methylation values for all individual CpGs covered in the unmodified primer assays by evaluating the sequenced data.

**Figure 2:**
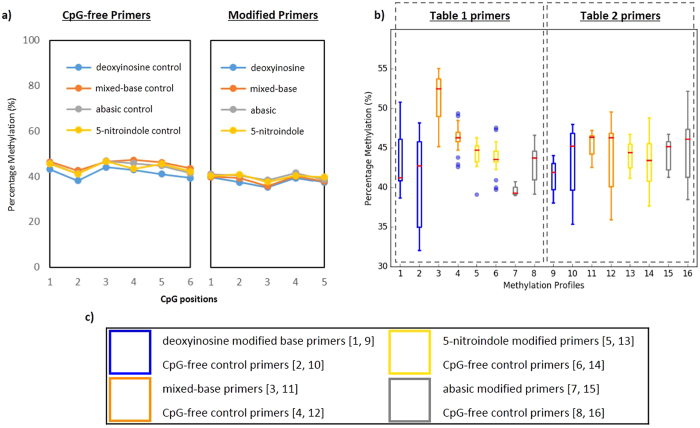
Representative bisulfite multiplex resequencing results of 50% methylated control sample using different modified primers. Sequencing results of representative libraries were prepared and sequenced using custom multiplex bisulfite resequencing. (**a**) Comparison of methylation profile of a representative amplicon (left) where a single CpG in the forward primer is substituted with one of the four modified bases), and CpG-free control (right), where amplicons were generated using CpG-free control primers produced a consistent level of methylation between the former and later indicating no prejudice in the multiplex reaction. (**b**) The portion of amplicons generated from primers with each modified base is within a similar range to the control amplicons used in each pool. (**c**) Modified bases corresponding to the box and whiskers plot in (**b**).

When the results of the unmodified primers from the deoxyinosine, mixed-base, dspacer and 5-nitroindole pools were assessed, a level of methylation was observed in line with the expected level based on the methylated control used (i.e. 100% 75%, 50%, 25 and 0%), and representative results for the 50% methylated control are shown in Fig. 2, and the mean and standard deviation of the different amplicons sequenced summarised in Supp Table 3. Moreover, the data obtained for the modified primers showed no demonstrable bias away from the mean values of the methylated controls. To further confirm that none of the modifications introduced bias a t-test assuming unequal variance was performed between the unmodified assays and those which contained modifications; no comparison reached the threshold of significance, indicating that the different modifications behaved similarly to the unmodified primer assays.

**Figure 3:**
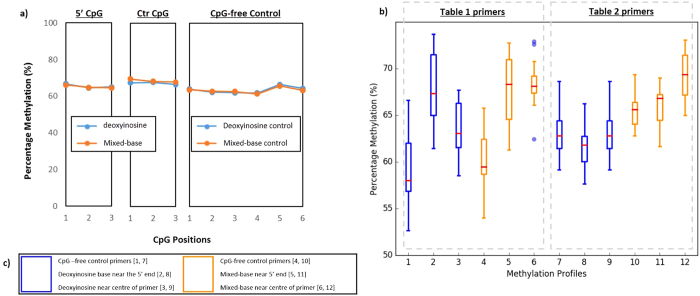
Representative sequencing results of 75% methylated control sample using deoxyinosine- and degenerate base primers. Sequencing results of representative libraries comparing the positional effects of deoxyinosine- and degenerate base modifications in modified primers. (**a**) Comparison of methylation profile of a representative amplicons, where the modification is located near the 5′ terminus of the forward primer (left panel, ‘5′CpG’), modification is located near the centre of the forward primer (middle panel, ‘Ctr CpG’), and CpG free control (right, ‘CpG-free Control). Comparison of the three sets of amplicons showed a minimal level of difference in methylation between the tree sets of amplicons. (**b**) Although the portion of amplicons generated from primers with the modification at either the 5′ end or centre of the forward primer appeared to be quite different when visualised, statistical analysis showed no significant difference between the two and the CpG-free control-generated amplicons. (**c**) Modified bases corresponding to the box and whiskers plot in (**b**), with corresponding boxplot shown in [ ].

To quantitatively determine if the position of the base modifications have any influence on methylation analysis in multiplex bisulfite PCR resequencing the data was then grouped by those primer pairs that had a modification towards the 5′ end of the primer, versus those where the modification was placed near the middle of the oligonucleotide. However, for this analysis only assays that had either a deoxyinosine or mixed-base modifications were used, since only a limited number of assays with either 5-nitroindole or abasic modifications passed the singleplex screen (Table 2, and Supp Figure 2). Results from this analysis indicated no demonstrable difference in the level of methylation between amplicons generated by the deoxyinosine and mixed-base assays, regardless of the location of the modification within the primer (representative data shown in Fig. 3 with a summary of the mean and standard deviation of the sequenced amplicons in Supp Table 4). This was further confirmed by performing a t-test assuming unequal variance, which presented no statistical difference between the modified assays and the unmodified controls.

**Figure 4:**
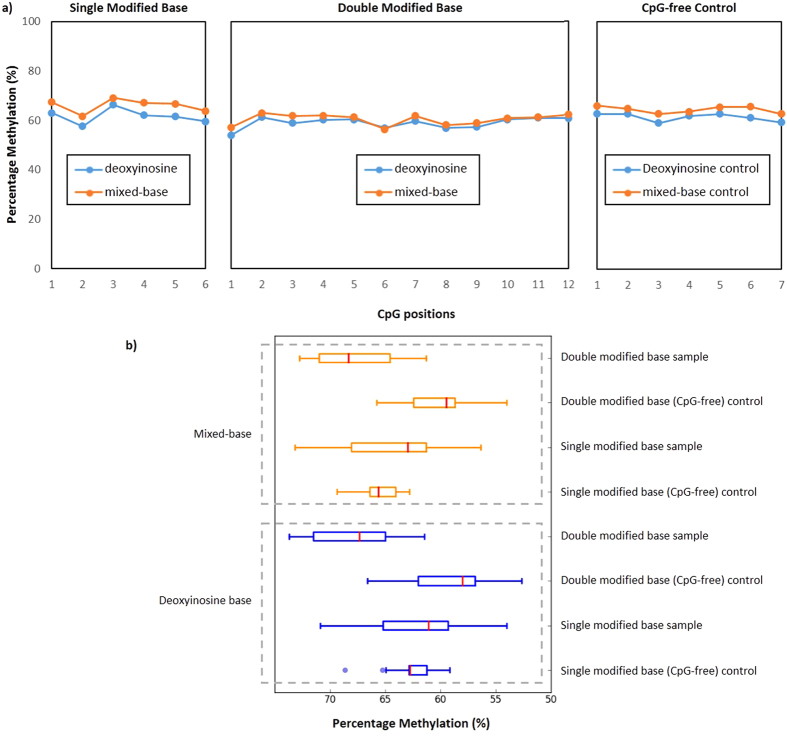
Representative result of amplicons generated using primers with either single or double CpG during bisulfite multiplex PCR. (**a**) Methylation profile of representative regions amplified with primers with a single CpG (a CpG is included in the forward primer), Double CpG (a CpG is included in both the forward and reverse primer), and CpG-free (no CpG) Control. When the C’s of the relevant CpGs were substituted with either degenerate or deoxyinosine bases, there was a similar level of methylation between the modified primers (primers with modified bases) and the CpG-free control. (**b**) The proportion of amplicons (of 75% methylated control) generated from primers pairs with either a single CpG or double CpG, and CpG-free controls compared using a box and whiskers plot.

To assess if the inclusion of a modification in both the forward and reverse primer led to a preferential amplification of methylated templates, the data for assays which had a modification in both the forward and reverse primer were compared to unmodified assays, as well as those in which only one primer was modified (modification located in the 5′ end of the forward primer). Similar to the previous analysis, only assays that had either a deoxyinosine or degenerate base modification were used and the results of this comparison indicated no demonstrable difference between the assay sets (representative region shown in Fig. 4 and a summary of the mean and standard deviation of the sequenced amplicons shown in Supp Table 4). This observation suggested the use of assays with double modifications has does not influence the level of methylation when screened using bisulfite multiplex PCR resequencing under the conditions used (primer concentration of 23 nM, PCR annealing temperature of 57 °C), and was further confirmed by statistical t-test assuming unequal variance, as no statistical difference was obtained when comparing the three conditions evaluated.

**Figure 5:**
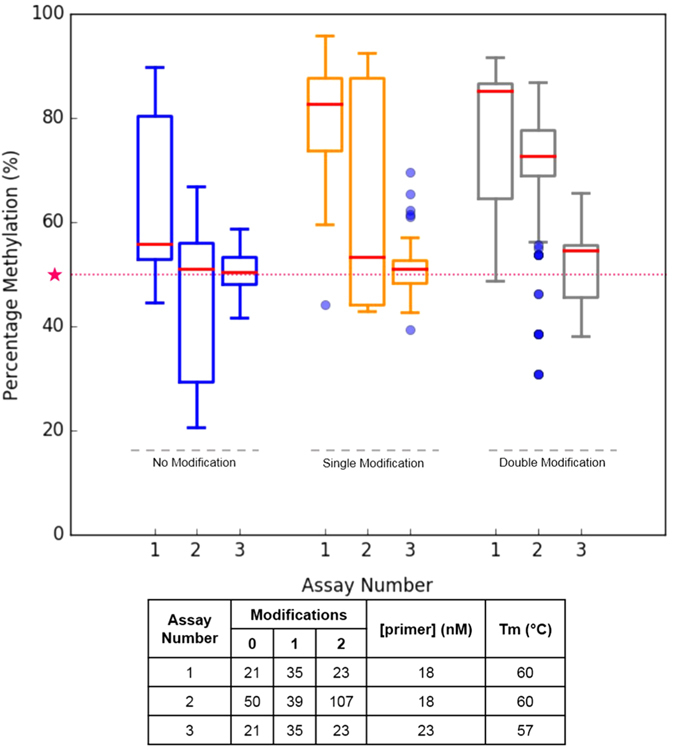
Representative result of amplicons generated using different conditions. The proportion of amplicons (of 50% methylated control) generated from assays with ‘no modifications’, ‘single modifications’ or double modifications. Baseline level of methylation (50%) is highlighted as ★. The number of amplicons in each assay and the corresponding primer concentration and melting temperature is as summarised in table. The [primer concentration] represents the concentration of the forward and reverse primer for every PCR assay in the multiplex pool.

To assess the conditions under which bias towards the methylated template could be induced, a larger set of 79 assays with modified bases were analysed in a large-scale multiple reaction. Notably, modified bisulfite PCR assays were designed using only mixed–base modifications, as these performed robustly and represented the most cost-effective solution. Assays were designed to have either a single or a double modification, and CpG-free primer pairs were also included as baseline controls. Multiplex amplification was then performed against five methylated controls (100% 75%, 50%, 25 and 0%) with two different annealing temperatures and primer concentrations (representative data shown in Fig. 5 with remaining results summarised in Supp Figure 3 and the mean and standard deviation of each data set presented in Supp Table 5). The results from this analysis indicated that a shift in amplification towards methylated templates could be induced using higher annealing temperatures and reducing the concentration of primer in the PCR assay, a trend which has also been observed in other studies^11^,^20^,^21^. Interestingly there was a significant shift in the mean from the optimal level of methylation in 0 and 75% methylated control (Supp Figure 3a, Supp Figure 3c and Supp Table 5), which is likely a result of the difference in annealing temperature of the bases in the mixed-base modifications used - i.e. in a Y base modification where there is a mix of cytosine and thymine, a higher annealing temperature will favour annealing of oligonucleotides containing cytosines at the mixed-base position, which could potentially skew methylation values towards the methylated transcript. In particular, as the 0% control is made up of whole genome amplified DNA, inefficiencies in template conversion and individual assay performances could lead to an increase in methylation pattern above the 0% baseline level of methylation (data not shown). From this, we conclude that the ideal reaction conditions for multiplex amplification are a 57 °C annealing temperature, and a minimum primer concentration of 23 nM. Under these conditions, bias towards the methylated transcript appears to be minimized.

## Conclusions

Bisulfite modification followed by PCR amplification allows the determination of methylation status of 5-methylcytosines at a single-nucleotide resolution, and some studies have proposed the inclusion of a limited number of CpGs in the primer when designing assays^20^,^21^. However, to date the effects of positioning a primer to bind a CpG site and including an oligonucleotide modification at the position of the 5-methylcytosines has not been evaluated in bisulfite multiplex PCR resequencing applications. In response, this study examined the effects of substituting different modifications at the cytosine of position of CpG dinucleotides contained within bisulfite PCR primers, and determined whether biased amplification towards the methylated template occurred.

The performance of four different oligonucleotide modifications assessed in this analysis were selected based on reports of their stable properties in other PCR-based studies. Mixed-base substitutions are often used when there is more than one base possibility at a particular position within a primer, and when using bisulfite primers a mixture of cytosine and thymine (denoted by the IUPAC base Y) and guanine or adenine (denoted by the IUPAC base R) were evaluated as substitutions at the cytosine position of CpG dinucleotides within PCR primers. The two different universal bases deoxyinosine and 5-nitroindole were also used, which can hybridise to all four possible DNA bases. Finally, primers with abasic sites was also evaluated, which in contrast to the previous three modified bases provides a space at the site of the modification. To ensure the data generated represents the minimum required for basic statistics we have endeavoured to ensure each modification is represented at least 3 times in our dataset. However, an increase in the number of representations could potentially improve the results of this study.

One unexpected result was that bisulfite PCR primers with either a 5-nitroindole or an abasic site did not result in reliable assays, and oligonucleotides with these modifications frequently failed to generate PCR product. This was surprising given that primers with 5-nitroindole had been reported to lead to improved performances in PCR due to its stabilizing properties under different conditions^13^,^19^,^22^. However, the different results obtained in our study with 5-nitroindole-modified primers could be due to the larger number of assays screened, compared to the original studies that were more restricted in their analysis^13^,^19^. Although the reason for inconsistent performance of dspacer-modified primers in our study was less clear, we hypothesised this could be due to the unstable nature of the modification at a higher annealing temperatures^23^.

In contrast, assays with either deoxyinosine or mixed-base primer modifications performed robustly under the conditions used. All primer pairs with these modifications consistently generated products of the right size in the singleplex bisulfite PCR screen, and methylation values obtained using these assays generated results comparable to the CpG-free controls. The position of the modified base in the primer did not seem to impact results, as modifications positioned near both the centre or the 5′ end of the assay produced comparable data to the unmodified controls. A similar conclusion was also made when comparing data from assays that contained a modification in both the forward and reverse primers, or only the forward primer.

Of note, a number of oligonucleotide suppliers (e.g. Integrated DNA Technologies) provides an option to either ‘machine-mix’ (default) or ‘hand-mix’ the proportions of nucleotides in mixed-base substitutions to achieve specific ratios for each base^14^, and the machine-mixed option was selected for this study. Judging from the results obtained, the default ‘machine-mix’ option does not skew the results in bisulfite multiplex sequencing as the methylation pattern of assays using the same primers with deoxyinosine modification were similar. Moreover, as ‘machine-mixed’ mixed-base assays are more cost-effective than the deoxyinosine modification and deliver similar performance, the use of mixed-based bisulfite PCR assays should represent the best option for many laboratories. However, it should be noted that a deoxyinosine-modified assay can be more effective than mixed-base primers as each deoxyinosine can theoretically bind to all templates in a reaction whereas mixed-base primers can only bind to a portion of the available template, the efficacy of which is dependent on the quantity of each base at the mixed-base site, and is altered after each amplification round. Therefore, if cost is not an issue we theorize that the performance of each assay could be potentially enhanced in some systems with the use of deoxyinosine.

Finally, an additional set of experiments was undertaken to assess the conditions under which preferential amplification towards the methylated template can be induced in multiplex bisulfite sequencing. Based on these results, a reduced annealing temperature of 57 °C and a higher primer concentration of 23 nM (when using assays with a salt-adjusted annealing temperature of 54 °C) was noted to generate data comparable to the CpG-free control assays. In contrast, the use of higher annealing temperatures and lower primer concentrations induced a substantial shift towards amplification of the methylated transcript. Although bias of this type is not ideal, it also provides a framework wherein low levels of methylated template may be selectively amplified away from a background of unmethylated DNA, leading to an increase in sensitivity of detection although at the cost of accurate methylation profiling. Therefore, care should be taken when employing modified assays of this type to fully optimise both the primer concentration and annealing temperature, as minor deviations can lead to preferential amplification of methylated templates and lead to bias in the outcome.

From our initial investigation, it was theorized that the same methodology could be applied to study non-CpG methylation in an unbiased context since non-CpG methylation detection also requires the availability of bisulfite-converted templates. Non-CpG methylation is found predominantly in non-somatic cells (accounting for <25% of the methylome of these target cells) and interests in this area has been gaining momentum in recent years due to its potential involvement in neuronal plasticity, neurogenesis and various neurological disorders^24^,^25^,^26^. Therefore, the use of modified primers could potentially be utilized in the study of non-CpG methylation in various contexts.

In conclusion, designing bisulfite PCR-based assays can be challenging due to the presence of CpG dinucleotides in the same region where primers are positioned. While the use of CpG-free primers may be considered the safest option to control for preferential amplification of methylated templates, this strategy is often challenging due to the CpG-rich nature of target regions. When CpG dinucleotides must be included in a primer-binding region, the use of primers with mixed-based or deoxyinosine substitutions can produce robust results in multiplex bisulfite PCR sequencing.

## Methods

### Modified Primer Design

Modified primer pairs were designed to have a CpG in the 5′ end of the forward primer, a CpG in the 5′ end of both the forward and reverse primers, or 0 CpGs in both primers. Primers were designed using our custom primer design software PrimerSuite (www.primer-suite.com.au)^27^ with the following parameters: oligo melting temperature (Tm) 54 °C, sodium concentration: 50 Mm, minimum CpGs in amplicon: 5, minimum number of unconverted bases from the 3′ end: 3. Each primer set was designed to have deoxyinosine (dI), mixed-base (Y (for primers targeting the sense strand) or R (primers targeting the anti-sense strand)), 5-nitroindole or abasic site in place of cytosine of the CpG. All primers were designed against breast cancer-associated regions described in Stirzaker *et al*.^28^ and outlined in Supp Table 1 and Supp Table 2. Fusion sequences were designed onto the 5′ end of both the forward and reverse primers (forward fusion sequence: ACACTGACGACATGGTTCTACAGA, reverse fusion sequence: TACGGTAGCAGAGACTTGGTCT).

### Methylated Controls

Five methylated control samples (i.e. 100%, 75%, 50%, 25%, 0%) were prepared by mixing a one hundred percent synthetically methylated DNA with a zero percent methylated human genomic DNA (created from whole genome amplification) according to protocols described in Korbie *et al*.^12^.

### Screening individual primer pairs using bisulfite PCR

Each primer pair was validated using bisulfite PCR following conditions outlined in Korbie *et al*.^12^. Briefly, genomic DNA extracted from white blood cells of different patients – ‘mixed blood pool’ was bisulfite converted prior to amplification using the following cycling conditions: 94 °C, 5 min; 12 cycles (95 °C, 20 s; 60 °C, 1 min); 12 cycles (94 °C, 20 s; 65 °C, 1 min 30 s); and 65 °C, 3 min, 10 °C hold. Visualisation of amplicons were performed using standard gel electrophoresis techniques with SB buffer at 200 V for 18 min using 1.5% agarose gel.

### Preparation and Sequencing of Modified Primer Libraries

Libraries of primer pairs which successfully amplified target amplicons during the singleplex assay were pooled and sequenced following protocols as described in Korbie *et al*.^12^. Briefly, primer pairs targeting different regions with the same base modifications were grouped into the same pool, and amplicons pooled in this way were differentiated from other pools using unique DNA barcodes (Fig. 1) to de-multiplex the data.

### Analysis of Modified Primer Data

Bioinformatic analysis of sequenced libraries were performed according to protocols outlined in Korbie *et al*.^12^. Briefly, adaptors of sequenced amplicons were trimmed using *Trim galore* (options: –length 100) prior to mapping to bisulfite-converted human genome using the *Bismark* methylation mapping program via *Bowtie2* (options: –bowtie2 -N 1 -L 15 –bam -p2 –score L, -0.6, -0.6 –non_directional; bismark_methylation_extractor –s merge_non_CpG –comprehensive –cytosine_report)^29^,^30^. Graphing was performed in both Excel 2010 and Python 3 (using the matplotlib module)^31^. Further statistical analysis were performed using the Statistica/JMP software.

## Additional Information

**How to cite this article:** Lu, J. *et al*. Evaluation of Different Oligonucleotide Base Substitutions at CpG Binding sites in Multiplex Bisulfite-PCR sequencing. *Sci. Rep.* **7**, 45096; doi: 10.1038/srep45096 (2017).

**Publisher's note:** Springer Nature remains neutral with regard to jurisdictional claims in published maps and institutional affiliations.

## Supplementary Material

Supplementary Information

## Figures and Tables

**Table 1:** Summary of singleplex bisulfite PCR results where the modified base is located in either a single primer or in both primers.

Forward Primer	Reverse Primer	Which primer was modified	Deoxyinosine	Mixed-base	5-nitroindole	Dspacer	CpG-free control
M201	M202	Neither	—	—	—	—	Positive
M203	M204	Neither	—	—	—	—	Positive
M205	M206	Neither	—	—	—	—	Positive
M209	M210	Forward	Positive	Positive	No amplification	No amplification	—
M213	M214	Forward	Positive	Positive	Positive	Positive	—
M215	M216	Forward	Positive	Positive	Positive	Positive	—
M217	M218	Both	Positive	Positive	No amplification^#^	No amplification^#^	—
M219	M220	Both	Positive	Positive	No amplification	No amplification	—
M221	M222	Both	Positive	Positive	No amplification	No amplification	—

^*^modifications are located in either the ‘forward’ or ‘both’ the forward and reverse primers. In each state, the modification is located near the 5′ end of the primer(s). In ‘CpG-free’ control primers, ‘Neither’ implied that that both the forward and reverse primers have no modifications. Primer sequences are supplied in SuppTable 1. ^#^Resultant product on gel was unclear and therefore excluded from further analysis.

**Table 2:** Summary of single-plex bisulfite PCR results where the modified base is located near the centre or 5′ end of the forward primer.

Forward Primer	Reverse Primer	Position of modification	Deoxyinosine	Mixed base	5-nitroindole	Dspacer	CpG-free control
C571	C572	None	—	—	—	—	Positive
C575	C576	None	—	—	—	—	Positive
C601	C602	Centre	Positive	Positive	No amplification	No amplification	—
C603	C604	Centre	Positive	Positive	No amplification	No amplification	—
C605	C606	Centre	Positive^$^	Positive^$^	No amplification	No amplification	—
C607	C608	Centre	Positive	Positive	No amplification	No amplification	—
C609	C610	Centre	Positive^$^	Positive^$^	No amplification	No amplification	—
C611	C612	5′	Positive	Positive	No amplification	No amplification	—
C613	C614	5′	Positive	Positive	Positive	Positive	—
C615	C616	5’	Positive	Positive	Positive	Positive	—
C617	C618	5′	Positive	Positive	No amplification	No amplification	—
C619	C620	5′	Positive	Positive	No amplification	No amplification	—

^*^Modified base is located near ‘centre’ or 5′ terminus of the forward primer, None is indicated for ‘CpG-free’ primer controls; ^$^Additional products seen on gel. Primer sequences are supplied in Supp Table 2.
