# Supplementary material for: Evaluation of Different Oligonucleotide Base Substitutions at CpG Binding sites in Multiplex Bisulfite-PCR sequencing
Source: Sci Rep. 2017 Mar 22;7:45096. doi: 10.1038/srep45096 (PMC5361148; doi:10.1038/srep45096)
Supplement: Supplementary Information [file srep45096-s1.pdf]

Evaluation of Different Oligonucleotide Base Substitutions at CpG Binding sites in Multiplex Bisulfite-PCR sequencing

Jennifer Lu, Kelin Ru, Ida Candiloro, Alexander Dobrovic, Darren Korbie, Matt Trau

SuppTable 1: Primers where the base modification is designed in either a single or both primers

Forward Primer Name*	Forward Primer Sequence*	Reverse Primer Name	Reverse Primer Sequence
M209_dI	5'-gGTTGGTATt-dI-gGtTttAGGG-3'	M210	5'-AaaACCTaaACATCCTaaaCCTT-3'
M209_Y	5'-gGTTGGTATtYgGtTttAGGG-3'		
M209_dS	5'-gGTTGGTATt-dS-gGtTttAGGG-3'		
M209_5Ni	5'-gGTTGGTATt-5Ni-gGtTttAGGG-3'		
M213_dI	5'-GAGt-dI-gAGATTTGGGtTtTGAAG-3'	M214	5'-TCTTTaCCACTaCTAAaATTACC-3'
M213_Y	5'-GAGtYgAGATTTGGGtTtTGAAG-3'		
M213_dS	5'-GAGt-dS-gAGATTTGGGtTtTGAAG-3'		
M213_5Ni	5'-GAGt-5Ni-gAGATTTGGGtTtTGAAG-3'		
M215_dI	5'-tAtTG-dI-gttttATtTGGAtTGAAATG-3'	M216	5'-TTaATCCTaCATCTaAACACCC-3'
M215_Y	5'-tAtTG-Y-gttttATtTGGAtTGAAATG-3'		
M215_dS	5'-tAtTG-dS-gttttATtTGGAtTGAAATG-3'		
M215_5Ni	5'-tAtTG-5Ni-gttttATtTGGAtTGAAATG-3'		
M217_dI	5'-TtTGA-dI-gttAAGGAGttAGttAGA-3'	M218_dI	5'-c-dI-TaTCCTCCCTTTaTTaTaaCTC-3'
M217_Y	5'-TtTGAYgttAAGGAGttAGttAGA-3'	M218_R	5'-cRTaTCCTCCCTTTaTTaTaaCTC-3'
M217_dS	5'-TtTGA-dS-gttAAGGAGttAGttAGA-3'	M218_dS	5'-c-dS-TaTCCTCCCTTTaTTaTaaCTC-3'
M217_5Ni	5'-TtTGA-5Ni-gttAAGGAGttAGttAGA-3'	M218_Ni	5'-c-5Ni-TaTCCTCCCTTTaTTaTaaCTC-3'
M219_dI	5'-tTAtttTGtttA-dI-gttAGATGGGG-3'	M220_dI	5'-c-dI-aAaaTaaaTaCACTCCACCTCT-3'
M219_Y	5'-tTAtttTGtttAYgttAGATGGGG-3'	M220_R	5'-cRaAaaTaaaTaCACTCCACCTCT-3'
M219_dS	5'-tTAtttTGtttA-dS-gttAGATGGGG-3'	M220_dS	5'-c-dS-aAaaTaaaTaCACTCCACCTCT-3'
M219_5Ni	5'-tTAtttTGtttA-5Ni-gttAGATGGGG-3'	M220_Ni	5'-c-5Ni-aAaaTaaaTaCACTCCACCTCT-3'
M221_dI	5'-GGtA-dI-gGGATGGAGTGAAAG-3'	M222_Di	5'-CCTCCCAATc-dI-AATCCCAAATT-3'
M221_Y	5'-GGtAYGggatggagtgaaag-3'	M222_R	5'-CCTCCCAATcRAATCCCAAATT-3'
M221_dS	5'-GGtA-dS-gGGATGGAGTGAAAG-3'	M222_dS	5'-CCTCCCAATc-dS-AATCCCAAATT-3'
M221_5Ni	5'-GGtA-5Ni-gGGATGGAGTGAAAG-3'	M222_Ni	5'-CCTCCCAATc-5Ni-AATCCCAAATT-3'
M201	5'-GAttAAGAtTtAGGGAAtATTGAGA-3'	M202	5'-TaCACCTCCCCAaCCCAT-3'
M203	5'-GATTGGATTGGGTTGtATTTGAA-3'	M204	5'-CCTCCTCTaaCCATaCATaTTT-3'
M205	5'-GGAAAGTTGAGGTAGAGtATG-3'	M206	5'-CTCCCTCCTCCCAaACAC-3'

*Modified base within the primer: dI – deoxyInosine, Y/R – mixed-bases (Y – C and T, R – G and A), dS – abasic, 5Ni – 5-Nitroindole

Evaluation of Different Oligonucleotide Base Substitutions at CpG Binding sites in Multiplex Bisulfite-PCR sequencing

Jennifer Lu, Kelin Ru, Ida Candiloro, Alexander Dobrovic, Darren Korbie, Matt Trau

SuppTable 2: Primers where the base modification is designed near either the 5' or the centre of the forward primer

Forward Primer Name*	Forward Primer Sequence*	Reverse Primer Name	Reverse Primer Sequence
C601_dI	5'-gGGTAGGG-dI-gGAGGTATT-3'	C602	5'-AaCTCTCCATTTaCTCTaAACA-3'
C601_Y	5'-gGGTAGGGYgGAGGTATT-3'		
C601_dS	5'-Gggtaggg-dS-gGAGGTATT-3'		
C601_5Ni	5'-gGGTAGGG-5Ni-gGAGGTATT-3'		
C603_dI	5'-AGGGGtTG-dI-gGGGAAAGG-3'	C604	5'-aTCAaCCCCTCTTTTaaaCTTC-3'
C603_Y	5'-AGGGGtTGYgGGGAAAGG-3'		
C603_dS	5'-AGGGGtTG-dS-gGGGAAAGG-3'		
C603_5Ni	5'-AGGGGtTG-5Ni-gGGGAAAGG-3'		
C605_dI	5’-AAGGGtAG-dI-gGGGTGATG-3’	C606	5’-TCACAaATaCCCACTTTCCC-3’
C605_	5’-AAGGGtAGYgGGGTGATG-3’		
C605_dS	5’-AAGGGtAG-dS-gGGGTGATG-3’		
C605_5Ni	5’-AAGGGtAG-5Ni-gGGGTGATG-3’		
C607_dI	5'-gGGAGTGG-dI-gAGGGTTTA-3'	C608	5'-CAaCCTCCCAaCTaaCCAC-3'
C607_Y	5'-gGGAGTGGYgAGGGTTTA-3'		
C607_dS	5'-gGGAGTGG-dS-gAGGGTTTA-3'		
C607_5Ni	5'-gGGAGTGG-5Ni-gAGGGTTTA-3'		
C609_dI	5’-GGAGAGtT-dI-gAGGGGAGG-3’	C610	5’-aTCCACCCCTCAaaTTCAC-3’
C609_Y	5’-GGAGAGtTYgAGGGGAGG-3’		
C609_dS	5’-GGAGAGtT-dS-gAGGGGAGG-3’		
C609_5Ni	5’-GGAGAGtT-5Ni-gAGGGGAGG-3’		
C611_dI	5'-CCTc-dI-TaCCAATAATTTAAACTTC-3'	C612	5'-ttAGGGTAGATGTGGGAAAG-3'
C611_R	5'-CCTcRTaCCAATAATTTAAACTTC-3'		
C611_dS	5'-CCTc-dS-TaCCAATAATTTAAACTTC-3'		
C611_5Ni	5'-CCTc-5Ni-TaCCAATAATTTAAACTTC-3'		
C613_dI	5'-TtAAT-dI-gTGTGGGtATGTTTtATTA-3'	C614	5'-CTTTaTTTTCCTTACCTTAAACAT-3'
C613_Y	5'-TtAATYgTGTGGGtATGTTTtATTA-3'		
C613_dS	5'-TtAAT-dS-gTGTGGGtATGTTTtATTA-3'		
C613_5Ni	5'-TtAAT-5Ni-gTGTGGGtATGTTTtATTA-3'		
C615_dI	5'-TCc-dI-aaaCTCCTaaAACCTTTAC-3'	C616	5'-ttTttAAGttAGtAGAAATttAGGA-3'
C615_R	5'-TCcRaaaCTCCTaaAACCTTTAC-3'		
C615_5Ni	5'-TCc-5Ni-aaaCTCCTaaAACCTTTAC-3'		
C615_dS	5'-TCc-dS-aaaCTCCTaaAACCTTTAC-3'		
C617_dI	5'-ACCTc-dI-aAaaAaCTTCTaCTAATTC-3'	C618	5'-TGGAAtttAGGTtTGTGAGAG-3'
C617_R	5'-ACCTcRaAaaAaCTTCTaCTAATTC-3'		
C617_dS	5'-ACCTc-dS-aAaaAaCTTCTaCTAATTC-3'		
C617_5Ni	5'-ACCTc-5Ni-aAaaAaCTTCTaCTAATTC-3'		
C619_dI	5'-CTCTc-dI-TaAACATTCAAaACAAAAC-3'	C620	5'-ttTTGtttATTTAGtTGAtTAGGAA-3'
C619_R	5'-CTCTcRTaAACATTCAAaACAAAAC-3'		
C619_dS	5'-CTCTc-dS-TaAACATTCAAaACAAAAC-3'		
C619_5Ni	5'-CTCTc-5Ni-TaAACATTCAAaACAAAAC-3'		
C379	5'-TaaaCTTTTaaTaaAaaAAaAaCAATCAT-3'	C380	5'-GGTGGTTGTGAAttAGGGA-3'
C383	5'-aCTaaaaaCCACAaCCCCAAC-3'	C384	5'-tTTGGGAtAAAGGtttAtTGAG-3'
C527	5'-AAtAtttAGtTGGAAGTTAATGAAT-3'	C528	5'-aCACTTCCACTCCCACC-3'

*Modified base within the primer: dI – deoxyInosine, Y/R – mixed-bases (Y – C and T, R – G and A), dS – abasic, 5Ni – 5-Nitroindole

SuppTable 3: Summary of mean and standard deviation (SD) of CpG methylation sites across all the amplicons generated with primers that had the four modified bases.

	deoxyinosine			mixed-base			5-nitroindole			abasic		
	**n***	**mean**	**SD**	**n***	**mean**	**SD**	**n***	**mean**	**SD**	**n***	**mean**	**SD**
**100% Methylated Control**												
**Modified Primers**	9	88.43	2.29	9	87.51	2.81	9	88.49	2.53	9	78.47	6.24
**CpG-free Control Primers**	23	86.65	3.11	23	85.66	3.24	23	85.38	4.05	23	84.94	5.00
**Modified Primers**	15	87.86	6.16	15	87.53	6.43	15	38.71	40.30	6	88.47	3.91
**CpG-free Control Primers**	15	87.18	3.30	15	87.43	3.64	15	87.86	4.46	15	86.71	4.45
**75% Methylated Control**												
**Modified Primers**	9	63.03	2.51	9	67.11	2.15	9	68.62	2.36	9	71.56	5.93
**CpG-free Control Primers**	23	59.19	3.56	23	60.26	2.71	23	62.94	2.87	23	63.14	3.20
**Modified Primers**	15	53.40	8.00	15	67.19	3.64	15	78.55	12.00	15	79.47	9.62
**CpG-free Control Primers**	15	60.70	4.03	15	64.22	2.97	15	66.32	5.33	15	67.89	4.70
**50% Methylated Control**												
**Modified Primers**	9	42.66	4.22	9	51.83	3.26	9	44.59	1.26	2	39.17	0.20
**CpG-free Control Primers**	23	41.03	5.44	23	46.28	1.66	23	43.74	1.93	23	43.24	2.24
**Modified Primers**	15	39.84	2.48	15	43.64	3.06	15	56.90	11.46	6	44.30	2.35
**CpG-free Control Primers**	15	43.47	4.30	15	44.11	4.19	15	43.61	3.32	15	45.42	4.36
**25% Methylated Control**												
**Modified Primers**	9	22.66	3.45	9	26.11	1.71	9	24.84	2.44	9	31.34	6.97
**CpG-free Control Primers**	23	21.86	1.44	23	18.64	3.23	23	23.16	1.31	23	24.51	1.54
**Modified Primers**	15	18.84	2.58	15	22.19	1.92	6	25.97	1.51	6	21.81	1.57
**CpG-free Control Primers**	15	22.40	3.30	15	19.82	1.82	15	22.81	1.64	15	22.54	2.33
**0% Methylated Control**												
**Modified Primers**	9	1.45	0.50	9	1.60	0.58	9	1.61	0.35	2	0.67	0.02
**CpG-free Control Primers**	23	1.42	0.59	23	1.53	0.63	23	1.61	0.78	23	1.60	0.49
**Modified Primers**	15	1.48	0.68	15	1.33	0.68	6	1.54	0.55	15	3.22	1.95
**CpG-free Control Primers**	15	1.42	0.42	15	1.27	0.33	15	1.34	0.40	15	1.82	0.66

***n**: number of CpG sites in total

SuppTable 4: Summary of mean and standard deviation (SD) of CpG methylation sites across amplicons generated using primers where the modified base is positioned differently within the primer(s).

	deoxyinosine			mixed-base		
	n*	mean	SD	n*	mean	SD
**100% Methylated Control**						
**CpG near 5' end**	8	88.25	3.61	8	87.87	3.93
**near centre of primer**	15	87.55	2.62	15	87.27	2.95
**CpG-free control**	23	86.65	3.11	23	85.66	3.24
**CpG near 5' end**	27	86.97	5.66	27	88.62	5.49
**CpG in both primers**	23	88.62	4.88	23	88.16	4.05
**CpG-free control**	15	87.18	3.30	15	87.43	3.64
**75% Methylated Control**						
**CpG near 5' end**	8	64.77	3.27	8	68.67	3.32
**near centre of primer**	15	65.07	3.89	15	71.00	8.42
**CpG-free control**	23	59.19	3.56	23	60.26	2.71
**CpG near 5' end**	27	50.38	7.20	27	67.83	3.30
**CpG in both primers**	23	62.15	4.43	23	64.01	4.60
**CpG-free control**	15	60.70	4.03	15	64.22	2.97
**50% Methylated Control**						
**CpG near 5' end**	8	44.17	1.89	8	46.30	2.04
**near centre of primer**	15	45.32	4.74	15	52.47	6.14
**CpG-free control**	23	41.03	5.44	23	46.28	1.66
**CpG near 5' end**	27	34.46	6.46	27	44.23	2.55
**CpG in both primers**	23	41.93	5.81	23	43.92	3.25
**CpG-free control**	15	43.47	4.30	15	44.11	4.19
**25% Methylated Control**						
**CpG near 5' end**	8	24.07	1.20	8	24.01	1.28
**near centre of primer**	15	25.27	4.36	15	26.00	1.49
**CpG-free control**	23	21.86	1.44	23	18.64	3.23
**CpG near 5' end**	27	15.24	4.57	27	23.96	2.51
**CpG in both primers**	23	18.30	3.62	23	21.39	5.04
**CpG-free control**	15	22.40	3.30	15	19.82	1.82
**0% Methylated Control**						
**CpG near 5' end**	8	1.59	0.48	8	1.19	0.26
**near centre of primer**	15	1.82	0.96	15	1.92	0.93
**CpG-free control**	23	1.42	0.59	23	1.53	0.63
**CpG near 5' end**	27	1.36	0.60	27	1.29	0.65
**CpG in both primers**	23	1.66	0.84	23	1.89	0.84
**CpG-free control**	15	1.42	0.42	15	1.27	0.33

***n**: number of CpG sites in total

SuppTable 5: Summary of mean and standard deviation (SD) of CpG methylation sites across different assay conditions.

		Percentage Methylation (%)				
		**100**	**75**	**50**	**25**	**0**
**No Modifications**						
**Assay 1 (n* = 21)**	**Mean**	96.48	79.96	62.24	30.37	15.05
	**SD**	1.05	11.28	16.38	12.72	9.20
**Assay 2 (n* = 51)**	**Mean**	97.55	63.87	46.68	15.00	11.73
	**SD**	1.67	15.55	14.91	5.61	6.88
**Assay 3 (n* = 22)**	**Mean**	97.40	72.16	50.27	24.22	11.95
	**SD**	1.00	6.10	5.21	3.70	5.18
**Single Modification**						
**Assay 1 (n* = 35)**	**Mean**	97.01	89.13	79.50	41.57	19.16
	**SD**	1.83	4.45	11.14	18.18	15.47
**Assay 2 (n* = 40)**	**Mean**	98.26	83.20	62.67	19.49	15.34
	**SD**	1.23	11.04	19.93	11.06	9.40
**Assay 3 (n* = 36)**	**Mean**	97.24	72.78	51.55	22.94	12.90
	**SD**	1.07	6.83	6.30	8.43	7.53
**Double Modification**						
**Assay 1 (n* = 23)**	**Mean**	97.04	89.60	76.90	56.42	18.74
	**SD**	1.24	9.72	16.82	26.78	10.85
**Assay 2 (n* = 108)**	**Mean**	97.96	88.33	70.35	28.54	18.88
	**SD**	1.75	4.61	13.28	16.55	15.11
**Assay 3 (n* = 24)**	**Mean**	97.94	70.62	51.43	25.84	15.75
	**SD**	0.76	9.73	7.59	9.31	7.08

***n**: number of CpG sites in total; Conditions of different assays are as summarised in SuppFigure 3

Evaluation of Different Oligonucleotide Base Substitutions at CpG Binding sites in Multiplex Bisulfite-PCR sequencing

Jennifer Lu, Kelin Ru, Ida Candiloro, Alexander Dobrovic, Darren Korbie, Matt Trau

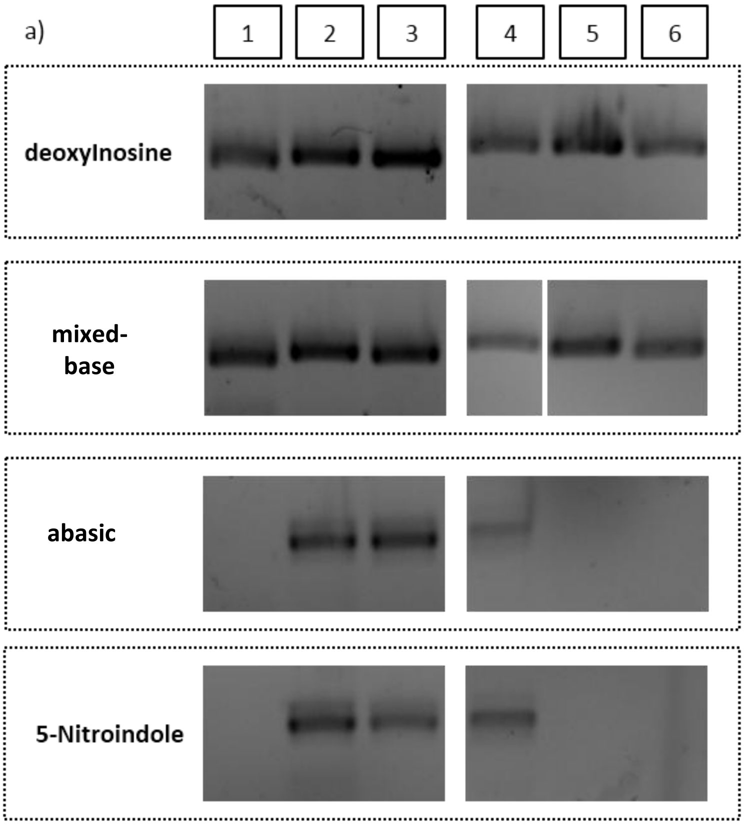

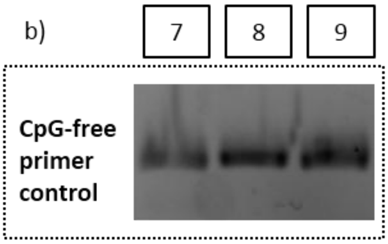

c) Table of primes used

Number	Forward Primer	Reverse Primer
1	M209	M210
2	M213	M214
3	M215	M216
4	M217	M218
5	M219	M220
6	M221	M222
7	M201	M202
8	M203	M204
9	M205	M206

SuppFigure 1: Validation of primer pairs performed using singleplex bisulfite PCR. a) Amplicons generated using primers where only one modification is designed in the forward primer (**lanes 1-3**) or a modification is designed in both the forward and reverse primer (**lanes 4-6**). While primer pairs with either deoxyinosine or mixed-base modifications produced amplicons regardless of the number of modifications, amplification using abasic- and 5-Nitroindole-modified primers were 'unpredictable'. b) amplicons generated using 'CpG-free' control primers indicated that the results produced in (a) were reliable and not as a result of foreign factors. **c**) Table of all the primers where the primers corresponding to the lane numbers in (**a**) and (**b**) are listed.

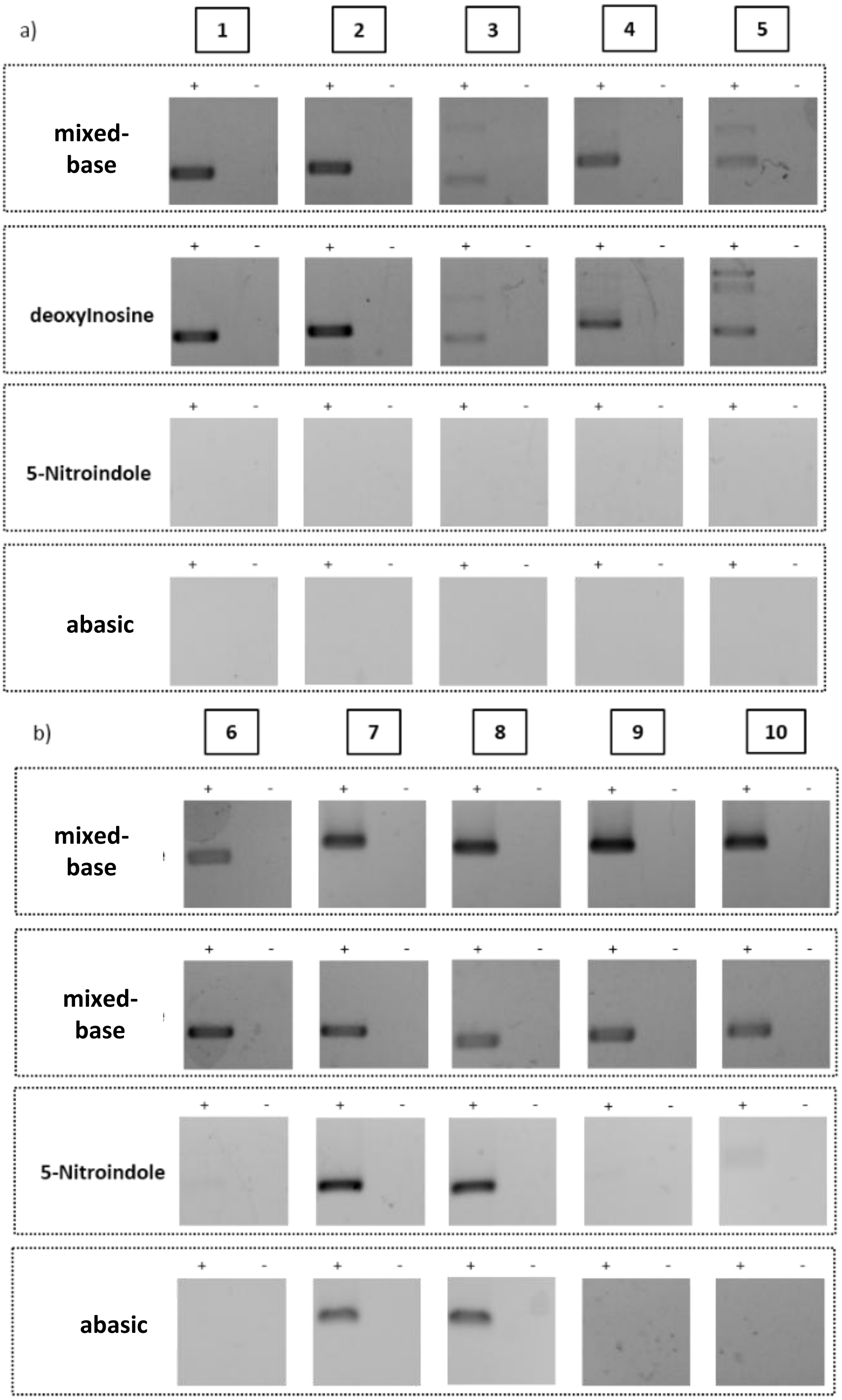

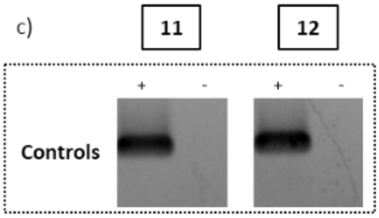

d) Table of primers used

Number	Forward Primer	Reverse Primer
1	C601	C602
2	C603	C604
3	C605	C606
4	C607	C608
5	C609	C610
6	C611	C612
7	C613	C614
8	C615	C616
9	C617	C618
10	C619	C620
11	C571	C572
12	C575	C576

SuppFigure 2: Validation of primer pairs using singleplex bisulfite PCR. a) Primer screen of primers where the modification is located near the centre of the forward primer. While primers with mixed-base of deoxyinosine modifications were able to produce products, primers with 5-nitroindole or abasic near the centre of the primer universally failed. **b)** Primer screen of primers where the modification is located near the 5' terminus of the forward primer. All primers which had a mixed-base or deoxyinosine modification near the 5' end of the forward primer produced products, while only two primer pairs with a 5-nitroindole or abasic near the 5' terminus produced amplicons, which suggests that the latter two modifications is not as stable as the former in bisulfite screening. **c)** Screening of 'CpG-free' primers during bisulfite PCR. All validation was performed on bisulfite-converted blood pool DNA. **d**) a table of all the primers where the primers corresponding to the lane numbers in (**a**), (**b**) and (**c**) are listed.

Evaluation of Different Oligonucleotide Base Substitutions at CpG Binding sites in Multiplex Bisulfite-PCR sequencing

Jennifer Lu, Kelin Ru, Ida Candiloro, Alexander Dobrovic, Darren Korbie, Matt Trau

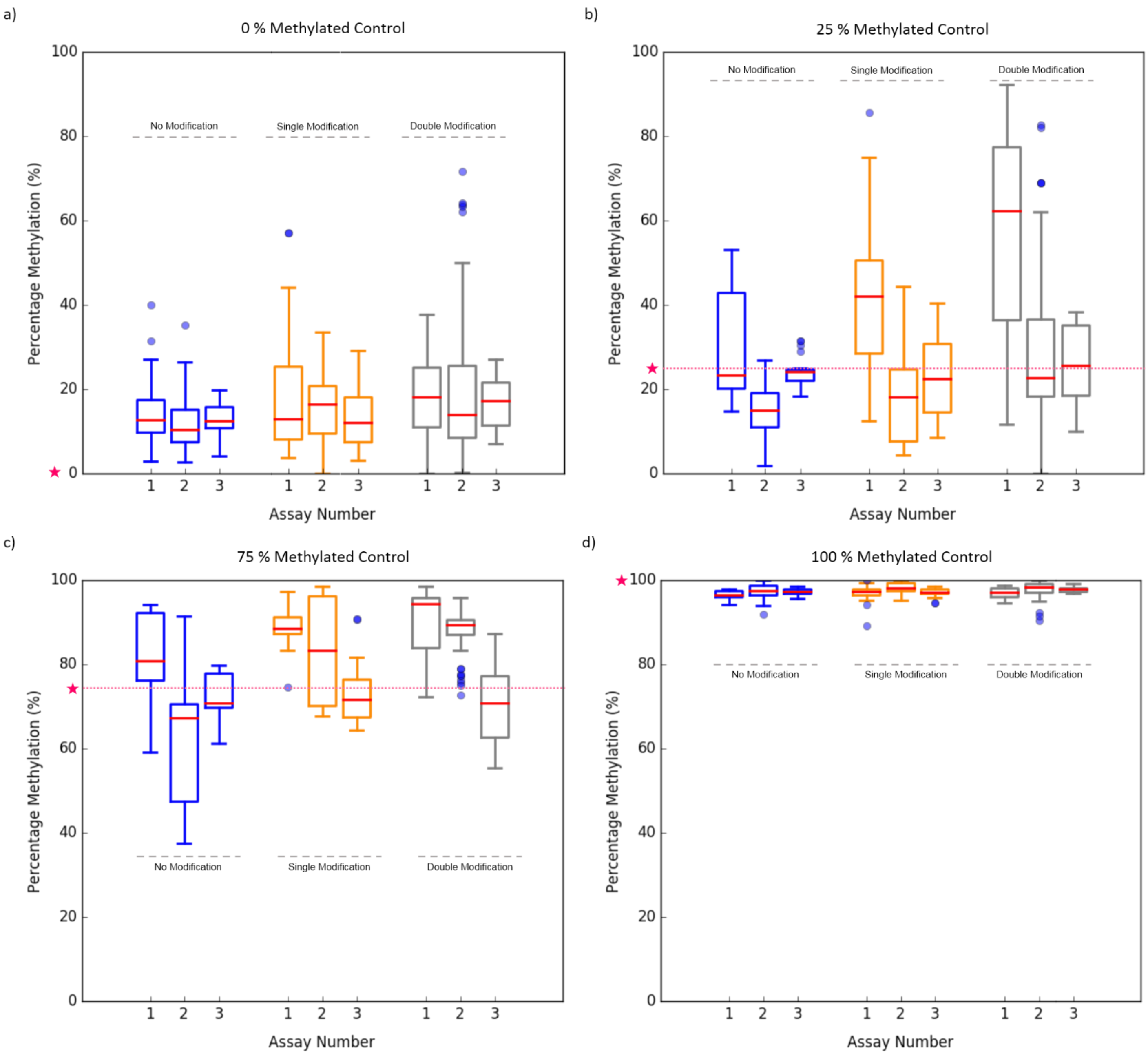

e)

Assay Number	Modifications			[primer] (nM)	Tm (°C)
	0	1	2		
1	21	35	23	18	60
2	50	39	107	18	60
3	21	35	23	23	57

SuppFigure 3: Summary of methylation analysis of different graduated methylation profiles using different assay conditions. Summary of multiplex bisulfite PCR screening of amplicons generated using primers with 'no modification' (both the forward and reverse primers have no CpG sites), 'single modification' (a single CpG site in either the forward or reverse primer) or double modification (a CpG site within both the forward and reverse primer) of four different graduated methylation controls (0%, 25%, 75% and 100%) shown in **a - d**. Optimal level of methylation for each methylated control is indicated with ★ along the y axis. e) Summary of assay conditions including primer concentrations (in nM) and annealing temperature (of the PCR).
